# Designing for Age-Inclusivity: A Cross-Disciplinary Approach to Landscape Architecture Education

**DOI:** 10.1093/geroni/igaf122.408

**Published:** 2025-12-31

**Authors:** Abigail Stephan, Thomas Schurch, Cordelia Wayt

**Affiliations:** Clemson University, Clemson, South Carolina, United States; Clemson University, Clemson, South Carolina, United States; Clemson University, Clemson, South Carolina, United States

## Abstract

As society’s proportion of older adults increases, professionals across disciplines must be aware of the opportunities and challenges associated with an aging population. Gerontology intersects with landscape architecture to promote cognitive, physical, and psychological well-being across the lifespan by enhancing access to nature. To prepare landscape architects-in-training to consider the impact of an aging population on their profession, our team developed a semester-long community design studio for second-year undergraduate landscape architecture majors to craft design plans for a local (1) 55+ townhome community and (2) residential care facility. Students engaged in project-based, experiential learning with scaffolded support that drew on the expertise of faculty, staff, and practitioners across disciplines (e.g., landscape architecture, gerontology, horticulture, environmental psychology, non-profit leadership). We conducted a mixed methods investigation to explore students’ (N = 10) experience in the studio and determine its influence on perceptions of aging, rated nature exposure importance across the lifespan, and understanding of their future career’s role in meeting societal challenges. Pre- and post-semester survey results revealed no statistically significant changes in students’ aging perceptions and perceived nature exposure importance. However, students’ accounts from focus group discussions suggest students enjoyed engaging with older adults and this experience was meaningful in advancing their understanding of the ways in which landscape architects play a critical role in shaping more nature-rich, age-friendly environments. Taken together, our findings demonstrate that the community design studio experience was valuable for students and led to a more nuanced perspective of the interdisciplinary approach required to successfully serve an aging society.

